# Overview of the safety, efficiency, and potential mechanisms of finerenone for diabetic kidney diseases

**DOI:** 10.3389/fendo.2023.1320603

**Published:** 2023-12-20

**Authors:** Wenmin Chen, Lingqian Zheng, Jiali Wang, Yongda Lin, Tianbiao Zhou

**Affiliations:** Department of Nephrology, The Second Affiliated Hospital of Shantou University Medical College, Shantou, China

**Keywords:** finerenone, chronic kidney disease, type 2 diabetes, diabetic kidney disease, cardiovascular disease, non-steroidal mineralocorticoid receptor antagonist, hyperkalemia, reactive oxygen species

## Abstract

Diabetic kidney disease (DKD) is a common disorder with numerous severe clinical implications. Due to a high level of fibrosis and inflammation that contributes to renal and cardiovascular disease (CVD), existing treatments have not effectively mitigated residual risk for patients with DKD. Excess activation of mineralocorticoid receptors (MRs) plays a significant role in the progression of renal and CVD, mostly by stimulating fibrosis and inflammation. However, the application of traditional steroidal MR antagonists (MRAs) to DKD has been limited by adverse events. Finerenone (FIN), a third-generation non-steroidal selective MRA, has revealed anti-fibrotic and anti-inflammatory effects in pre-clinical studies. Current clinical trials, such as FIDELIO-DKD and FIGARO-DKD and their combined analysis FIDELITY, have elucidated that FIN reduces the kidney and CV composite outcomes and risk of hyperkalemia compared to traditional steroidal MRAs in patients with DKD. As a result, FIN should be regarded as one of the mainstays of treatment for patients with DKD. In this review, the safety, efficiency, and potential mechanisms of FIN treatment on the renal system in patients with DKD is reviewed.

## 1 Introduction

Chronic kidney disease (CKD) is a significant global public health challenge characterized by persistent abnormalities in kidney structure or function such as albuminuria, abnormal urinary sediment and decreased estimated glomerular filtration rate (eGFR) for at least three months, with associated symptomology (1, 2). It is a major contributor to morbidity and mortality on a world scale, with a reported prevalence of 9.1% in 2017 (3). CKD is anticipated to rank as the fifth-leading cause of death around the world by 2040 and the second-cause of death before the end of the century in countries with extended life expectancies (4). Over 850 million individuals worldwide have CKD, acute kidney injury (AKI), and are receiving renal replacement therapy (5). The prevalence has been increasing with serious and significant implications for public health and society attributed to disease burden, complications leading to cardiovascular (CV) morbidity, excess mortality, and costs associated with managing kidney failure (3).

Among a broad range of etiologies, diabetes has emerged as the primary cause of CKD globally, with an grown risk of disease progression, CV events, and mortality (6, 7). About 40% of individuals diagnosed with type 2 diabetes (T2D) are susceptible to the development of diabetic kidney disease (DKD), a condition that may progress to end-stage kidney disease (ESKD) and subsequently contribute to the burden of CV disease (CVD) (8–13). The mortality rate of DKD exceeds that of diabetes alone or CKD without diabetes by more than two-fold (14, 15). The coexistence of diabetes and CKD has been demonstrated to shorten the average life expectancy by about 16 years, representing a major challenge for society and public health systems all over the world (14). Therefore, new strategies that not only protect the kidney but also reduce the risk of CV events development should be imperatively taken into account in patients with DKD.

The development and progression of CKD in individuals with T2D are influenced by various factors, including hemodynamic factors, metabolic factors, and mineralocorticoid receptor (MR) overactivation (9, 16). In the kidney, MR is expressed in the distal tubules, collecting ducts, podocytes, fibroblasts, and mesangial cells (17). Upregulation of MR is evident in several clinical conditions such as hyperglycemia, CKD, albuminuria, cardiac disease, and high salt (HS) intake (18–24). MR overarousal promotes oxidative stress, inflammation, and fibrosis, resulting in renal alterations such as changes in the sodium-potassium ATPase in the distal convoluted tubule, sodium retention, elevated blood pressure (BP), glomerular hypertrophy, glomerulosclerosis, mesangial proliferation, and tubulointerstitial fibrosis, and ultimately contributing to the progression of CKD and CV complication (13, 17, 25–33). Hence, early intervention and intensive treatment are essential to mitigate renal and CV complications in individuals with DKD (34).

While renin-angiotensin system (RAS) blockers (e.g., angiotensin-converting enzyme inhibitors (ACEI), angiotensin receptor blockers (ARB)) and sodium-glucose co-transporters-2 inhibitors (SGLT2i) have shown beneficial renal and CV effects in DKD patients by targeting hemodynamic and metabolic drivers of CKD progression (35–40), they inadequately address the inflammation and fibrosis driven by MR overactivation, leading to a persistent high residual risk of CKD progression and CV events development, even in response to combined treatment by these two therapies (9, 13, 37, 38, 41). Therefore, a comprehensive approach is imperative to address the broader pathogenesis of DKD patients, including increased fibrosis and inflammation (25, 32, 42–44). Given this context, it is evident that MR antagonists (MRAs) play a pivotal role in the prevention of fibrosis and inflammation in both the renal and CV systems.

At the renal level, MR blockage significantly reduces albuminuria and promotes the preservation of renal function (32, 45–47). In the past years, classical steroidal MRAs like first-generation spironolactone and second-generation eplerenone, while potentially beneficial for nephroprotection and cardioprotection, are hindered by the risk of hyperkalemia and other progestogenic and antiandrogenic adverse effects (AEs) such as breast tenderness, gynecomastia, erectile dysfunction in men, and menstrual irregularities in premenopausal women, particularly in patients with DKD (48–55). The risk of hyperkalemia can escalate up to 8-fold in patients with moderate-to-severe CKD using steroidal MRAs (53, 56). While these AEs are not typically life-threatening, they can undermine treatment adherence and persistence, with roughly half of the patients discontinuing MRAs and 10% of patients continuing at reduced dose due to hyperkalemia (54). The conundrum of possessing effective therapies but not employing them due to safety concerns has prompted substantial efforts over the past two decades to develop novel MRAs with improved safety profiles.

Now, the emergence of the nonsteroidal MRAs (NS-MRAs) with an improved benefit-risk profile, exemplified by finerenone (FIN), offers a new opportunity for MRAs in DKD (57). To overcome the inherent limitations of steroidal MRAs by achieving high MR specificity and a balanced distribution between cardiac and renal tissues, FIN, a novel, nonsteroidal, selective, and potent third-generation MRA with enhanced antifibrotic and anti-inflammatory properties and a reduced incidence of hyperkalemia compared to traditional MRAs, is currently the most studied and has received approved for the treatment of DKD (58–61). An array of preclinical and clinical studies has substantiated the efficacy and safety of FIN in conferring renal and CV benefits.

On these grounds, this review aims to elucidate the molecular mechanisms of FIN and provide insights into its efficacy and safety across the spectrum of DKD patients, including those with and without a history of CVD.

## 2 Physiological and pathophysiological roles of MR in the kidney

The primary physiological role of the MR, found in the epithelial cells of the kidney and colon, is to regulate water and electrolyte balance (62). However, MR is also present in non-epithelial cells within the kidney and various extrarenal tissues, including the heart and vasculature (63). Upregulation of MR in these non-epithelial cells in the heart and kidney leads to increased transcription of profibrotic genes such as transforming growth factor-β-1 (TGF-β1), connective tissue growth factor, plasminogen activator inhibitor-1 (PAI-1), and various extracellular matrix proteins including fibronectin and collagens, all of which are associated with renal and cardiac fibrosis (33). Additionally, there is a positive correlation between elevated levels of serum aldosterone and an increased susceptibility to renal failure in both diabetic and non-diabetic individuals (64). Accumulating evidence indicates that the activation of MR is linked to injury in podocytes through various mechanisms. These mechanisms include the involvement of Ras-related C3 botulinum toxin substrate 1 (Rac1), the reduction of autophagy which is crucial for podocyte maintenance, and an increase in NADPH oxidases (NOX) activity resulting in oxidative stress and further leads to the upregulation of a cascade of proinflammatory cytokines and profibrotic proteins (65). Subsequently, development of albuminuria, reduced renal blood flow, and AKI lead to the progression of chronic renal interstitial inflammation and fibrosis (65, 66). Thus, MRAs may potentially delay the progression of CKD irrespective of its underlying cause (64). Accordingly, although blocking MR in non-epithelial cells has a positive impact, blockade of MR in epithelial cells increases the risk of hyperkalemia. The contrasting roles of MR in physiological and pathobiological processes need careful consideration of their interplay when implementing medication.

## 3 Effect of FIN on renal reactive oxygen species, inflammation and fibrosis

### 3.1 Renal ROS

**Figure 1:**
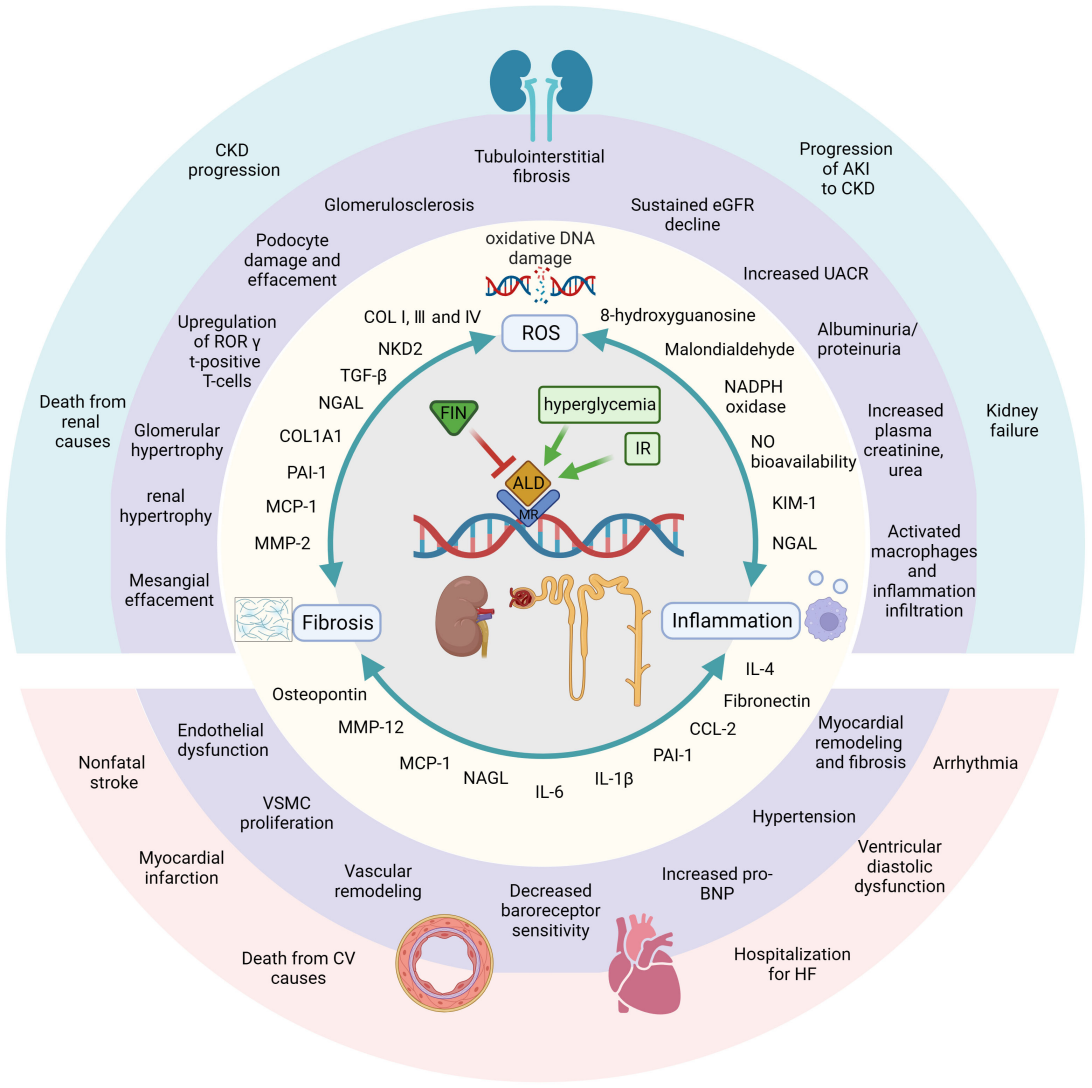
FIN displays beneficial effects against DKD and renal IR damage by different mechanisms of action in kidney. Diabetic and ischemic environment induce hyperactivation of MR and trigger three pathways, which can start a great variety of molecular, cellular, tissue and subsequently organ responses. On contrast, MRA FIN blocks the binding of aldosterone and MR, then blocks and attenuates those pathophysiological progressions. First, FIN alleviate oxidative stress by attenuating oxidative DNA damage and reducing the production of ROSs. The second major mechanism, FIN reduce the upregulation of pro-inflammatory mediators, leading to reduced inflammation. FIN also leads to reduced fibrosis by downregulating fibrotic biomarkers. In these ways, FIN shows renal-cardiovascular protective effect. Created by BioRender.com.

In the renal context, the overactivation of MR leads to an increased presence of ROS through the upregulation of NOX (67–69). These superoxide radicals have the potential to disrupt the normal functioning of both the renal vasculature and tubules. Additionally, hydrogen peroxide, another byproduct of this process, contributes to dysfunction, particularly in the preglomerular region (67–69). Nitric oxide (NO) bioavailability and increased oxidant damage are linked to ischemia in renal IR injury-inducing AKI (70). The generation of oxidative stress is decreased by the pharmacologic use of FIN or the genetic removal of MR in smooth muscle cells (SMCs) (71). In both mice (71) and rats (72), FIN has been demonstrated to suppress the expression of markers of tubular injury in the kidney, such as kidney injury molecule 1 (KIM-1) and neutrophil gelatinase-associated lipocalin (NGAL) (**Table 1**). It has also been shown that after renal IR damage, FIN normalizes pathophysiologic elevations in the oxidative stress markers like malondialdehyde and 8-hydroxyguanosine (**Figure 1**) (72).

**Table 1:** Efficacy of FIN for renal protection in animal experiments.

Author, Year	Animal Species	Modeling Type	Research Results
Kolkhof et al., 2014 (62)	Sprague-Dawley rats and Wistar rats	A rat model of DOCA/Salt-induced Heart and Kidney Injury	FIN ↓: proteinuria, glomerular tubular and vascular damage, risk of electrolyte disturbances, cardiac hypertrophy, BNP, renal expression of pro-inflammatory and pro-fibrotic markers (PAI-1, MCP-1, OPN, and MMP-2); FIN ↑: end-organ protection, systolic and diastolic left ventricular function
Barrera-Chimal et al., 2017 (65)	Male C57Bl/6 mice and Large White pig	Model of AKI induced by IR	FIN ↓: renal injury induced by IR through effects on Rac1-mediated MR signaling; renal mRNA levels of NGAL and Kim-1; oxidative stress production
Lattenist et al., 2017 (64)	Male Wistar rats	A rat model of AKI to CKD	FIN ↓: inflammatory factors; fibrogenic markers; oxidative stress markers; deposition of perinephric macrophages and collagen; proteinuria; tubulointerstitial fibrosis; acute injury induced by IR and the chronic and progressive deterioration of kidney function and structure
Barrera-Chimal et al., 2018 (27)	Male C57Bl/6 mice	A mice model of bilateral IR-induced CKD	FIN ↓: inflammatory population of CD11bD, F4/80D, Ly6Chigh macrophages; proinflammatory cytokines IL-6 and IL-1ß; subsequent chronic dysfunction and fibrosis induced by IR; FIN ↑: M2-antiinflamatory markers, IL-4 receptor.
González-Blázquez et al., 2018 (45)	Male Wistar and MWF rats	A genetic model of CKD	FIN ↓: albuminuria, endothelial dysfunction, superoxide anion levels; FIN ↑: NO bioavailability, SOD activity
Gil-Ortega et al., 2020 (46)	Male Wistar and MWF rats	A genetic model of CKD	FIN ↓: albuminuria, plasma MMP-2 and MMP-9 activities, superoxide anion levels, intrinsic (mesenteric) arterial stiffness; FIN ↑: plasma pro-MMP-2 activity, NO bioavailability
Droebner et al., 2021 (73)	C57BL/6J mice	2 relevant mouse kidney fibrosis models: unilateral ureter obstruction and sub-chronic IR injury	FIN has direct anti-fibrotic properties resulting in reduced myofibroblast and collagen deposition accompanied by a reduction in renal PAI-1 and NKD2 expression in mouse models of progressive kidney fibrosis at BP-independent dosages
Hirohama et al., 2021 (66)	Mice	Uninephrectomized T2M db/db mice fed a HS diet (DKD with hypertension model)	FIN ↓: Salt-induced activation of Rac1-MR pathway associated with Sgk1 upregulation and subsequent increased expressions of cleaved α-ENaC and phosphorylated NCC in distal tubules and glomeruli. FIN ↓: fibronectin; inflammatory markers (MCP-1 and PAI-1)
Kolkhof et al., 2021 (73)	Transgenic (mRen2)27 rats	A rat model of hypertension-induced end-organ damage	Combination of FIN and empagliflozin at low dosages effectively reduces cardiac and renal lesions, proteinuria, BP, creatinine, uric acid and mortality. FIN reduced renal fibrosis and the renal expression of pro-fibrotic COL1A1
Luettges et al., 2022 (63)	Male C57BL6/J mice	UNX DOCA-salt model	FIN protects against functional and morphological renal damage and exerts antihypertensive actions. FIN reduces renal IL-17 producing RORγt γδ T cells
Lima-Posada et al., 2023 (67)	ZSF1 rats	A model of DN associated with cardiac dysfunction	FIN did not impact kidney function but ↓ renal hypertrophy

### 3.2 Renal inflammation

In a murine knockout model of glomerulonephritis, it was observed that signaling of the MR in myeloid cells contributes to the progression of renal injury (29). One possible protective effect of MR knockout on myeloid cells against renal damage is a reduction in the recruitment of neutrophils and macrophages (29). The decrease in leukocytes was associated with the downregulation of pro-inflammatory markers’ gene expression, such as tumor necrosis factor-α (TNF-α), matrix metalloproteinase (MMP)-12, inducible NO synthase, and C-C motif chemokine ligand 2 (CCL-2) (29). Recruitment of macrophages is crucial during both the injury and repair phases after a kidney ischemia event (74). Activation of MR in monocytes tends to polarize macrophages toward an “inflammatory M1” phenotype (75). The rationale for utilizing MRAs to impede the progression from AKI to CKD is supported by the fact that MR inhibition via FIN promotes increased expression of interleukin (IL)-4 receptor in murine kidney IR models, subsequently facilitating the polarization of macrophage toward an M2 phenotype (27). This shift is accompanied by decreased macrophage mRNA expression of the pro-inflammatory cytokine TNF-α and the M1 macrophage marker IL-1β (27). Additionally, there is a reduction in the inflammatory population of CD11b^+^, F4/80^+^, and Ly6C^high^ macrophages (27). In uninephrectomized (UNX) DOCA-treated mice, the downregulation of kidney retinoid-related orphan receptor (ROR) gamma t-positive T-cells, along with a significant reduction in UACR, demonstrates significant renal protection in response to FIN treatment (76). Notably, MR antagonism by FIN can modulate inflammation as indicated by its ability to reduce proinflammatory cytokines like IL-6 and IL-1ß following renal ischemic damage (27). Furthermore, FIN has been shown to lower the expression of renal NGAL (71, 72), which is released during systemic inflammation by neutrophils and in response to tubular injury by renal tubular cells (71, 72). The pro-inflammatory cytokine monocyte chemoattractant protein-1 (MCP-1) is also reduced by FIN in the DOCA-salt model of cardiorenal end-organ damage (77). Both NGAL and MCP-1 play significant roles in the progression of human CKD (78, 79). Renal osteopontin (OPN) expression was also decreased in a DOCA-salt rat CKD model following FIN treatment (77). OPN is believed to regulate various aspects of renal fibrogenesis, including fibroblast proliferation, macrophage activation and infiltration, cytokine secretion, and extracellular matrix production. It is associated with CKD progression, with elevated plasma levels detected in the early stages of CKD (80). FIN also offers protection against podocyte damage in a murine model of CKD progression in T2D (UNX mice with T2D fed a HS diet), as indicated by reduced production of fibronectin and inflammatory markers such as MCP-1 and PAI-1 in glomeruli (**Figure 1**) (81).

### 3.3 Renal fibrosis

The development of kidney fibrosis is a critical factor in the progression of CKD and eventual renal failure, as it disrupts the structural integrity of renal tubules and adjacent blood vessels. Studies conducted on individuals with kidney diseases have identified pro-fibrotic cytokines like TGF-β, MCP-1, and MMP-2 as potential biomarkers for fibrosis development, which have been correlated with worsening renal function (WRF) (82). Additionally, plasma levels of PAI-1 have showed moderate correlations with fibrosis observed in biopsies (82). To investigate the role of MR in fibrosis development and CKD progression, as well as the effectiveness of FIN in mitigating renal fibrosis, various preclinical models have been employed. In the DOCA-salt rat model of CKD, FIN treatment led to a reduction in renal mRNA expression of the pro-fibrotic marker PAI-1 and a decrease in renal fibrosis as evaluated through histopathology (77). The study revealed that the administration of FIN had a dose-dependent effect in diminishing the upregulation of mRNA expression of MMP-2, which serves as a significant indicator of tissue remodeling (77). In a rat model of hypertensive cardiorenal disease, FIN also attenuated renal fibrosis and reduced the production of pro-fibrotic collagen type I α 1 chain (COL1A1) in the kidney (83). Moreover, FIN dose-dependently suppressed pathologic myofibroblast accumulation and collagen deposition in a mouse model of renal fibrosis, irrespective of changes in systemic blood pressure or inflammatory markers (84). The levels of the fibrotic biomarkers PAI-1 and naked cuticle homolog 2 (NKD2) were concomitantly decreased in the kidneys (84), with recent reports identifying NKD2 as a specific marker for myofibroblasts in human renal fibrosis (85). In a chronic CKD rat model characterized by renal dysfunction, elevated proteinuria, and extensive tubule-interstitial fibrosis, treatment with FIN effectively limited collagen deposition and fibrosis in the kidney, as confirmed through histopathological assessments (72). FIN also inhibited the upregulation of the pro-fibrotic cytokine TGF-β and collagen-I expression in the kidneys (72). Likewise, in a mouse CKD model featuring unilateral, IR-induced tubulo-interstitial fibrosis, FIN administration resulted in a significant reduction in the severity of renal fibrosis (27) (**Figure 1** and **Table 1**).

## 4 Renal protection of FIN in animal models

The efficacy of FIN for renal protection in animal experiments has been evaluated in recent years (**Table 1**). Kolkhof et al. (77) found that FIN consistently protects from functional as well as structural end-organ damage in kidneys with a reduced risk of electrolyte disturbances in the 10-week rat deoxycorticosterone acetate (DOCA)/salt model in a dose-dependent manner, with the most significant effect at 10 mg/kg·d. Histological analyses indicated that when compared at equinatriuretic doses, FIN outperformed eplerenone in reducing proteinuria and alleviating glomerular, tubular, and vascular damage (77). Similarly, Luettges et al. (76) conducted experiments highlighting FIN’s protective effects against functional and morphological renal damage and its ability to exert antihypertensive actions in mice subjected to the DOCA-Salt model. In a rodent model transitioning from AKI to CKD, FIN demonstrated efficacy in preventing AKI induced by ischemia-reperfusion (IR) and the subsequent chronic and progressive deterioration of kidney function and structure (27, 71, 72). These long-term protective effects of FIN were also observed in a preclinical model involving large white pigs (71). Furthermore, prophylactic FIN administration efficiently prevented increased plasma creatinine, urea, and proteinuria (27, 71, 72). Current investigations have unveiled the favorable impact of FIN on both arterial distensibility and albuminuria in the munich Wistar frömter (MWF) CKD model (45, 46). In uninephretomized db/db mice fed a HS diet, Hirohama et al. (81) reported that FIN ameliorated albuminuria, associated with reduced BP and glomerular injury. Nevertheless, in ZSF1 rats with diabetes, the administration of FIN did not significantly affect kidney function but did reduce renal hypertrophy (86).

## 5 Renal protection of FIN in clinic

**Figure 2:**
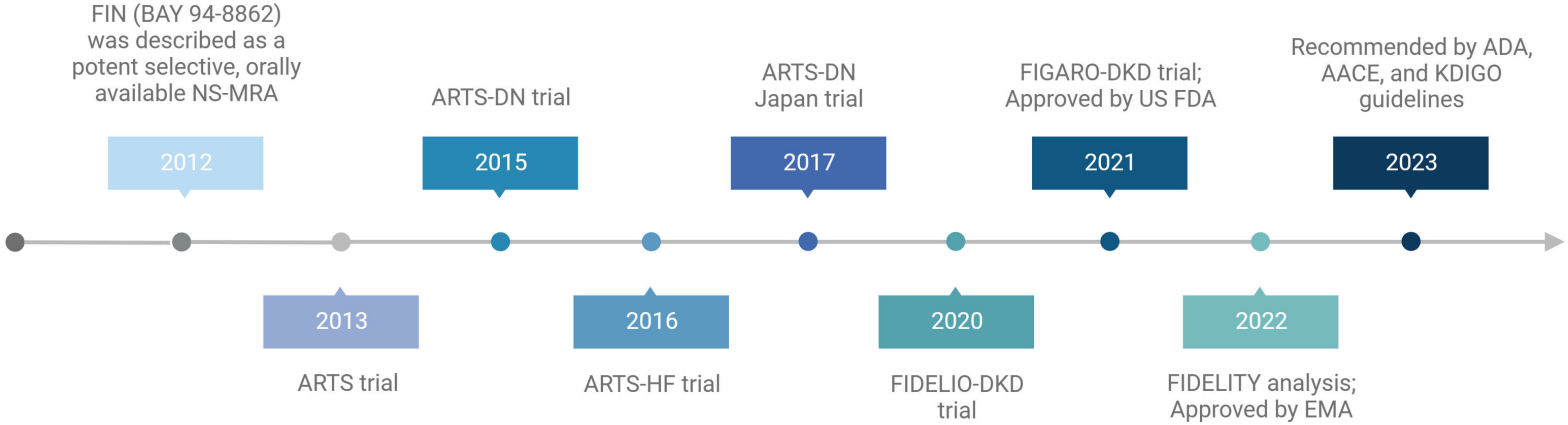
Summary of the milestones and main trials of finerenone. Created by BioRender.com.

The efficacy and safety profiles of FIN were evaluated in 4 phase II trials in patients with CVD and kidney disease within the ARTS program and 2 phase III trials in patients with DKD (**Table 2**) (**Figure 2**) (87–92).

**Table 2:** Efficacy of FIN for renal protection in clinic.

Trial, Auther, Year	Research Type	Phase	Race	Country	Sample Size	Patient’s Condition	Intervention	Duration	Change in End Indicators	Safety Evaluation
ARTS, Pitt et al., 2013 (70)	Randomized control study	IIa	/	Multi-center	part A, n = 65; part B, n = 392	HFrEF (NYHA II-III, LVEF≤40%) and mild or moderate CKD (eGFR 60–90 mL/min/1.73 m^2^ in the safety assessment [part A], or 30–60 mL/ min/1.73 m^2^ in the efficacy assessment [part B])	FIN (2.5, 5, or 10 mg QD [parts A & B], or 5 mg BID [part B]) Open-label spironolactone (25 or 50 mg/day [part B]) Placebo (parts A & B)	28 days (parts A & B)	FIN vs. spironolactone: equivalent or greater reductions in albuminuria and N-terminal pro-BNP levels; smaller decrease in eGFR	FIN vs. spironolactone: smaller increases in serum potassium concentration; lower incidences of hyperkalemia and WRF
ARTS-DN, Bakris et al., 2015 (72)	Randomized control study	IIb	White, Black or African American, Asian, Multiple, Not reported, Hispanic or Latino	Multi-center	823	DKD receiving RAS inhibitors (UACR ≥30 mg/g and eGFR >30 mL/min/1.73 m^2^)	FIN (1.25–20 mg/ day; 7 doses assessed) Placebo	90 days	Reduction in UACR: dose dependent at the 4 highest doses of FIN vs. placebo; 38% UACR reduction with FIN 20 mg/ day; Only a modest reduction in BP at the highest dosage of FIN	Hyperkalemia leading to discontinuation: not observed in the placebo and FIN 10 mg/ day groups; incidences in the FIN 7.5-20 mg/day groups were low (1.7%–3.2%). No differences in the incidence of an eGFR decrease of ≥30% or in incidences of AEs and serious AEs between the placebo and FIN groups
ARTS-HF, Filippatos et al., 2016 (71)	Randomized control study	IIb	Europe, North America, Asia, Other	Multi-center	1066	HFrEF and T2D and/or CKD (eGFR >30 mL/min/ 1.73 m^2^ in patients with diabetes or 30–60 mL/ min/1.73 m^2^ in those without diabetes)	FIN (2.5–15 mg/ day, uptitrated to 5-20 mg on day 30) Eplerenone (25 mg every other day, increased to 25 mg/day and 50 mg/day on days 30 and 60, respectively)	90 days	FIN vs. eplerenone: similar effects on N-terminal pro-BNP levels and BP; greater reduction of composite clinical outcome of all-cause death, CV hospitalization, or emergency presentation due to worsening HF	FIN vs. eplerenone: smaller increases in serum potassium concentration; similar incidence of hyperkalemic events and TEAEs
ARTS-DN Japan, Katayama et al., 2017 (74)	Randomized control study	IIb	Japanese	Multi-center	96	Japanese patients with DKD receiving a RAS blocker	FIN or placebo	90 days	The UACR at day 90 relative to baseline for each FIN treatment group was numerically reduced compared with placebo	No serious AEs or deaths were reported and no patients experienced TEAEs resulting in discontinuation of study drug. Small mean increases in serum potassium level in FIN vs. Placebo. No patients developed hyperkalemia
FIDELIO-DKD, Bakris et al., 2020 (69)	Randomized control study	III	White, Black/African American, Asian, Other	Multi-center	5734	DKD receiving RAS inhibitors First set: UACR 30 to <300 mg/g, eGFR 25 to <60 mL/ min/1.73 m^2^, and history of diabetic retinopathy. Second set: UACR 300–5000 mg/g, eGFR 25 to <75 mL/ min/1.73 m^2^	FIN (10–20 mg/ day). Placebo	2.6 years (median follow up)	Key primary composite outcome events: significantly less with FIN vs. placebo. Key secondary composite outcome events: significantly less with FIN vs. placebo. FIN was associated with a 31% reduction in the UACR from baseline to 4 months, and a lower UACR was maintained throughout the remainder of the trial.	Frequency of AEs: overall similar with FIN vs. placebo. Incidence of serum potassium levels >5.5 mmol/l: FIN (21.7%) vs. placebo (9.8%); The incidence of hyperkalemia-related AEs: FIN (18.3%) vs. placebo (9.0%); Hyperkalemia leading to discontinuation: FIN (2.3%) vs. placebo (0.9%); Serious hyperkalemia related AEs requiring hospitalization: FIN (1.4%) vs. placebo (0.3%); No report of any mortal event directly attributable to hyperkalemia
FIGARO-DKD, Pitt et al., 2021 (68)	Randomized control study	III	White, Black/African American, Asian, Other	Multi-center	7437	DKD receiving RAS inhibitors. First set: UACR 30 to <300 mg/ g, eGFR 25–90 mL/ min/1.73 m^2^. Second set: UACR 300-5000 mg/g, eGFR ≥60 mL/min/1.73 m^2^	FIN (10–20 mg/ day). Placebo	3.4 years (median follow up)	Key primary composite outcome events: significantly less with FIN vs. placebo; Key secondary composite outcome events: similar with FIN vs. placebo.	Frequency of AEs: overall similar with FIN vs. placebo; Hyperkalemia leading to discontinuation: higher with FIN than with placebo (1.2% vs. 0.4%)
FIDELITY, Agarwal et al., 2022 (76)	FIDELITY prespecified pooled analysis of FIDELIO-DKD and FIGARO-DKD	III	White, Black/African American, Asian, Other	Multi-center	13026	DKD	standard therapy + FIN or placebo	interquartile range 2.3–3.8 years	Significant reductions of 14% for the endpoint CV outcome; 23% for risk of kidney composite events; and 20% for dialysis initiation with a 20% reduction in ESKD and a 22% reduction in HF hospitalizations; and significantly reduced UACR by 32% compared to placebo.	AEs related to treatment: FIN (18.5%) vs. placebo (13.3%); AEs leading to treatment discontinuation FIN (6.4%) vs. placebo (5.4%); hyperkalemia: FIN (14%) vs. placebo (6.9%); Hospitalizations due to hyperkalemia FIN (0.9%) vs. placebo (0.2%); Hyperkalemia leading to permanent treatment discontinuation FIN (1.7%) vs. placebo (0.6%); No hyperkalemia-related AEs were fatal; The incidence of TEAE is similar in the FIN and placebo groups, with no increase in sex hormone-related AEs compared to the placebo group

### 5.1 Clinical efficacy of FIN treatment for patients with CKD and T2D

The Mineralocorticoid Receptor Antagonist Tolerability Study–Diabetic Nephropathy (ARTS-DN), a pioneering multicenter, phase II clinical trial investigating the use of FIN in combination with a RAS inhibitor in individuals with DKD exhibited significant reductions in albuminuria and enhancements in the urine albumin-creatinine ratio (UACR) after 90 days of treatment with FIN (7.5–20 mg/day) in comparison to a placebo (91). The results of the ARTS-DN Japan trial, which included 96 Japanese patients with DKD receiving a RAS inhibitor, support those of ARTS-DN (92). Detailed dose–exposure–response modeling and simulation, encompassing an analysis of both ARTS-DN and ARTS-DN Japan, suggested that the effects of FIN were predominantly saturated at a dosage of 20 mg, with both 10 mg and 20 mg administered once daily proving to be safe and effective in reducing albuminuria (93). Furthermore, there were no discernible differences in the incidence of a 30% decrease in eGFR between the treatment groups (91).

Certainly, the most comprehensive insights into the advantages of FIN in individuals with DKD have been garnered through pivotal phase III clinical trials: FIDELIO-DKD (Finerenone in Reducing Kidney Failure and Disease Progression in Diabetic Kidney Disease) (88); FIGARO-DKD (Finerenone in Reducing Cardiovascular Mortality and Morbidity in Diabetic Kidney Disease) (87); and the predefined combined analysis known as FIDELITY (Combined FIDELIO-DKD and FIGARO-DKD Trial program analysis) (94).

FIDELIO-DKD, a multicenter, double-blind randomized controlled trial (RCT), has furnished the initial clinical evidence affirming that MR blockade yields improvements in kidney outcomes for patients with DKD (88). This study included 5734 adults with DKD who were already receiving the maximum tolerated doses of an ACEI or ARB and maintained a serum potassium concentration of 4.8 mmol/L (88). Eligible patients had a UACR 30–<300 mg/g, an eGFR of 25–<60 ml/min/1.73 m^2^, and diabetic retinopathy, or they had a UACR of 300–5000 mg/g and an eGFR of 25–<75 ml/min/1.73 m^2^ (88). Following a median follow-up of 2.6 years, the results of the FIDELIO-DKD study exhibited a significantly lower incidence of the primary composite outcome, encompassing kidney failure, a sustained eGFR decline of ≥40% from baseline, or death from renal causes in the FIN group compared to the placebo group (17.8% vs. 21.1%) (88). At the 4-month post-treatment mark, UACR demonstrated a 31% reduction compared to the placebo group, with this difference persisting throughout the trial (88). A secondary model-based analysis of FIDELIO-DKD showed that the early UACR effect of FIN was predictive of its long-term impact on eGFR decline, and these effects were found to be independent of the concurrent use of SGLT2i (95). Furthermore, FIN achieved a placebo-subtracted reduction of 14% in the crucial secondary composite outcome, encompassing CV death, nonfatal myocardial infarction (MI), nonfatal stroke, or hospitalization for heart failure (HF) (88). In a secondary analysis of the FIDELIO-DKD trial, FIN was shown to reduce the risk of newly diagnosed atrial fibrillation/flutter compared to placebo, irrespective of a history of atrial arrhythmias at baseline (3.2% vs. 4.5%) (96).

Similarly, in the next major phase III FIGARO-DKD clinical trial (87), which enrolled 7437 adults with DKD across a wider range of CKD stages (a UACR of 30–<300 mg/g and an eGFR of 25–90 mL/min/1.73 m^2^ or a UACR of 300–5000 mg/g and an eGFR of≥60 ml/min/1.73 m^2^) already treated with maximum tolerated doses of an ACEI or ARB and maintaining a serum potassium concentration less than 4.8 mmol/L, a notable 13% reduction in the composite primary outcome, comprising CV death, nonfatal stroke, nonfatal MI, or hospitalization for HF, was observed after a mean follow-up of 3.4 years (87). While a lower incidence rate for the eGFR ≥40% kidney composite endpoint was noted with FIN compared to placebo, it did not reach statistical significance (P = 0.069). Nevertheless, a greater treatment effect was noted on the eGFR ≥57% kidney composite endpoint, with a 36% relative risk reduction for ESKD (P = 0.041) (87). FIN achieved a 32% greater reduction in UACR from baseline to 4 months compared to placebo (87), and its impact on kidney outcomes was particularly pronounced in patients with significantly elevated albuminuria as opposed to those with moderately increased albuminuria (97). An analysis derived from the FIGARO-DKD study emphasizes the importance of albuminuria screening in T2D patients, as early initiation of treatment effectively mitigated the risk of CV events and albuminuria progression in individuals with moderately elevated albuminuria (98).

The outcomes of the FIDELITY study, which pooled data from FIDELIO-DKD and FIGARO-DKD involving over 13,000 patients, revealed significant reductions associated with FIN, including a 23% reduction in the risk of kidney composite events (renal failure, >57% eGFR reduction, and renal disease-related death), a 20% reduction in dialysis initiation, a 32% reduction in UACR from baseline to 4 months, a 14% reduction in the primary composite CV outcome, and a 22% reduction in HF hospitalizations compared to placebo (94). Moreover, it’s worth emphasizing that FIN induced a modest reduction in mean systolic BP at 4 months (3.2mmHg vs. 0.5 mmHg increase with placebo) in the FIDELITY pooled analysis (94). While it did not exhibit significant differences between groups in the whole population from both FIDELIO-DKD and FIGARO-DKD, a FIDELITY *post hoc* analysis of a subgroup of patients with treatment-resistant hypertension showed a significant differences of the least squares mean change in office systolic BP between FIN and placebo groups during the first 4 months (−7.1 mmHg vs. −1.3 mmHg, respectively) (P <.0001) (99). Meanwhile, the cardiorenal effects of FIN do not seem to be primarily mediated by its antihypertensive properties, as the impact on BP was minimal compared to spironolactone (94, 99). There is no evidence suggesting a correlation between the antialbuminuric effects of FIN and changes in BP.

Further analyses demonstrate that FIN provides robust renal and CV efficacy and safety benefits across the spectrum of DKD (100, 101). FIN consistently showed improvements in indicators of kidney injury, as evidenced by a reduction in UACR and function, with better preservation of eGFR in the chronic phase, compared to placebo in patients with stage 4 CKD (101). However, the effect of FIN on the composite kidney outcome in patients with stage 4 CKD exhibited inconsistencies between early and late years of follow-up, with a notable loss of precision over time (101). A recent study included nine patients with advanced DKD with an eGFR below 25 mL/min/1.73 m^2^ revealed that FIN caused a significantly slower decline in eGFR in patients with advanced DKD, thus providing initial evidence that FIN is effective across a wide range of renal functions (102). As such, further large-scale investigation will be necessary to confirm the efficacy and safety of FIN in DKD patients with an eGFR below 25 mL/min/1.73 m^2^.

Furthermore, some FIDELITY analyses emphasize the benefits of early treatment initiation and co-administration of potassium-binding agents to maximize the protective effects of FIN in individuals with DKD (100, 103). FIN improved cardiorenal outcome in patients with DKD, regardless of baseline HbA1c (94, 104, 105), HbA1c variability (104), diabetes duration (104), baseline insulin use (104, 105), baseline HF history (106, 107), prevalent atherosclerotic CVD (108), and history of atrial fibrillation/flutter at baseline (96). Additionally, it is worth noting that the antihypertensive effect of adding FIN to a maximally tolerated dose of ACEI or ARB was relatively modest (87, 88).

In conclusion, these results suggest that in patients with DKD, FIN may be an effective treatment for kidney and CV protection. In fact, FIN has been approved by the U.S. Food and Drug Administration (FDA) in 2021 to reduce the risk of sustained eGFR decline, ESKD, nonfatal MI, hospitalization for HF, and CV death in adults with DKD (109). In addition, the European Medicines Agency (EMA) authorized the marketing of FIN for routine clinical use in patients with DKD on 16 February 2022 (110). Recently published guidelines from the American Diabetes Association (ADA) (111–113), the American Association of Clinical Endocrinology (AACE) (114) and the updated Kidney Disease: Improving Global Outcomes (KDIGO) Diabetes Work Group (112, 115) recommend the addition of the oral NS-MRAs FIN to standard treatment in patients with DKD (**Figure 2**).

### 5.2 Clinical efficacy of FIN treatment for patients with CVD and kidney disease

The phase IIa study known as Mineralocorticoid Receptor Antagonist Tolerability Study (ARTS) (89) represents the inaugural RCT of FIN. It encompassed both a double-blind placebo group and an open-label spironolactone comparison group and targeted individuals with heart failure with reduced ejection fraction (HFrEF) along with mild or moderate CKD. This study established safe dosing of FIN and demonstrated that FIN exhibited equivalent or greater reductions in albuminuria and N-terminal pro-B-type natri (89) uretic peptide (pro-BNP) levels compared to spironolactone. The study results indicated that individuals receiving FIN (10 mg q.d.) showed a significant smaller decrease in eGFR than those receiving spironolactone at visit 7 (day 29 + 2) (-2.69 vs. -6.70 ml/min/1.73 m^2^) (P ¼ 0.0002–0.0133) (89).

In the phase IIb ARTS-Heart Failure (ARTS-HF) study (90), which included 1066 patients diagnosed with HFrEF and T2D and/or CKD, there was a notable indication of benefit associated with FIN. Specifically, there was a significant reduction in the composite clinical outcome, which included events such as all-cause mortality, CV hospitalization, or emergency admissions due to worsening HF, among patients treated with FIN in comparison to those receiving eplerenone. A recent prespecified analysis of FIGARO-DKD indicated that FIN reduced the risk of developing HF independent of a history of HF (107). These findings suggest that FIN may offer valuable prospects as a treatment option for patients with heart failure with preserved ejection fraction (HFpEF), particularly those who also have T2D and/or CKD.

## 6 The safety of FIN treatment

Even though FIN has beneficial effects on renal outcomes, the safety of FIN therapy is important and is an essential precondition for clinical application. AEs associated with using FIN include the risk of hyperkalemia, renal insufficiency and sex hormone-associated AEs.

The safety profile of FIN underwent thorough investigation within an extensive phase II clinical trial program, encompassing over 2000 patients who had HFrEF, CKD, and/or T2D or with DKD (**Table 2**) (89–91). In the ARTS study, it was evident that the mean rise in serum potassium levels over a 28-day period was significantly lower in all four dosage groups of FIN when compared to the spironolactone group (0.04 to 0.30 vs. 0.45 mmol/L, respectively) and there was lower incidence of hyperkalemia with FIN vs spironolactone (5.3% vs 12.7%, p = 0.048) (89). In the ARTS-HF study, the mean increase in serum potassium from baseline to Day 90 was greater in the eplerenone group than in the FIN groups (90). Hyperkalemia, defined as a serum potassium elevation exceeding 5.6 mmol/L, was observed in 4.3% of patients, with the incidence of hyperkalemic events and treatment-emergent AEs (TEAEs) being similar in both the FIN and eplerenone groups (90). Within the ARTS-DN trial, permanent discontinuation of the medication due to hyperkalemia was not reported in the placebo group or the FIN 10 mg/day group. However, it occurred in 2.1%, 3.2%, and 1.7% of patients randomized to the FIN 7.5 mg/day, 15 mg/day, and 20 mg/day groups, respectively (91). Secondary safety outcomes, including a decline in eGFR of ≥30% and the incidence of other serious AEs, did not exhibit significant differences between the various treatment groups (91). In summary, these phase II trials collectively demonstrate that hyperkalemia does not serve as a substantial impediment to the utilization of FIN for renal protection (89–91).

With respect to the safety profile in the FIDELIO-DKD phase III trial (88), it is noteworthy that FIN treatment was generally well-tolerated, and the distribution of AEs was comparable between the FIN and placebo groups. It is worth mentioning that the incidence of serum potassium levels exceeding 5.5 mmol/L was higher in the FIN group compared to the placebo group (21.7% vs. 9.8%), and hyperkalemia-related AEs occurred at a double rate in FIN-treated patients compared to those receiving placebo (18.3% vs. 9.0%). However, it’s important to highlight that severe hyperkalemia-related AEs necessitating hospitalization were infrequent (1.4% vs. 0.3%), and there were no reported fatalities directly attributed to hyperkalemia. Permanent discontinuation of the medication was observed in 2.3% of patients in the FIN group and 0.9% in the placebo group. A secondary model-based analysis of FIDELIO-DKD revealed that higher FIN doses were linked to lower serum potassium levels and reduced incidences of hyperkalemia, guided by serum potassium-based dose adjustments (116). Additionally, FIN exhibited a lower risk of AEs related to sex hormones (117). In the FIGARO-DKD trial (87), the incidence of overall AEs and the risk of serious AEs leading to discontinuation were similar between the FIN and placebo groups. Although the occurrence of severe hyperkalemia was slightly higher with FIN than placebo (0.7% vs. 0.1%), it is noteworthy that no fatal hyperkalemia events were reported. The discontinuation rate due to hyperkalemia was also higher with FIN compared to placebo (1.2% vs. 0.4%, respectively). However, none of the hyperkalemia-related AEs resulted in fatalities. In the FIDELITY pooled analysis (94), 18.5% of patients who got FIN experienced treatment-related AEs, compared to 13.3% of patients who received a placebo, with AEs leading to treatment discontinuation occurring in 6.4% vs. 5.4% of patients, respectively. Hyperkalemia was more common with FIN than with placebo (14% vs. 6.9%). Hospitalizations due to hyperkalemia (0.9% and 0.2%, respectively) were minimal, and the incidence of hyperkalemia leading to permanent treatment discontinuation was low across study arms but occurred more frequently with FIN (1.7%) than with placebo (0.6%). However, no hyperkalemia-related AEs were fatal. The incidence of TEAEs was comparable between the FIN and placebo groups, and there were no notable increases in AEs related to sex hormones or AKI compared to the placebo group. Regarding patients with CKD stage 4, the safety profile of FIN in individuals with T2D remained consistent with that of CKD stages 1 to 3. A FIDELITY *post hoc* analysis showed that FIN was related to a lower risk of hyperkalemia compared with spironolactone with/without a potassium-binding agent (99).

In conclusion, FIN appeared safe and effective in most clinical studies (**Table 2**). Nevertheless, baseline eGFR and serum potassium levels should be evaluated before initiation of FIN, and periodic measurement of serum potassium should still be performed during treatment with FIN, and the dose adjusted as needed. Additionally, implementing dietary interventions, avoiding agents that have the potential to induce hyperkalemia (118), correcting metabolic acidosis, and using potassium-lowering drugs (119) are effective strategies for preventing hyperkalemia.

## 7 Combination treatment with ACEI/ARB and SGLT2i/glucagon-like peptide-1 receptor agonists

A subgroup analysis, based on various MRAs, indicates that the relative risk of hyperkalemia when combining ACEI/ARB with FIN is lower compared to eplerenone or spironolactone (120).

Additionally, the combination of FIN with a SGLT2i like empagliflozin at low dose provides renal protection effect and effectively reduces proteinuria, plasma creatinine, uric acid, BP, cardiac and renal lesions, and mortality in a nondiabetic hypertensive cardiorenal disease model (50). However, multiple clinical studies, including FIDELIGO-DKD, have illustrated that FIN alone reduces UACR independently of SGLT2i (121). In the FIDELITY analyses, FIN outperformed placebo in terms of cardiorenal outcomes in individuals with DKD, regardless of SGLT2i usage (73, 94). In other words, SGLT2i did not alter the effects of FIN on the primary endpoint. However, as for the safety, MRAs increase serum potassium concentration and the risk of hyperkalemia while SGLT2is reduce the risk of hyperkalemia (122, 123), which makes the combination of SGLT2i with MRAs an attractive treatment option from a safety perspective. Analysis from the FIDELIO-DKD trial reveals that when combined with FIN, treatment with an SGLT2i may offer protection from hyperkalemia events despite low number of hyperkalemia events was observed (73, 117). Moreover, it should be pointed out that in several trials, the SGLT2i dapagliflozin and MRA eplerenone reduce albuminuria and the incidence of hyperkalemia was significantly less during treatment with dapagliflozin-eplerenone compared with eplerenone alone (124, 125). These findings offer a convincing reason for evaluating the long-term efficacy and safety of combined SGLT2i and MRA treatment and may make eplerenone, a second-generation MRA, combined with SGLT2i, a second option for patients who can’t tolerate FIN or can’t afford such an expensive drug price of FIN.

Regarding GLP-1RAs, a *post hoc* exploratory analysis of the FIDELIO-DKD and FIGARO-DKD trials found that FIN reduces UACR in patients, irrespective of whether they were using GLP-1RAs use at the beginning of the study. The effects on kidney and CV outcomes remain consistent regardless of GLP-1RA usage, with no obvious safety signals associated with the combination treatment (126, 127).

In summary, the results from clinical studies comparing combination therapy to monotherapy vary. Understanding the molecular mechanisms and potential interactions between FIN, ACEI/ARB, and SGLT2i/GLP-1RA agents remains unclear. Consequently, further clinical trials and in-depth mechanistic research are essential to provide conclusive evidence.

## 8 Ongoing trials

So far, clinical investigation into the kidney and CV disease outcomes associated with FIN have primarily focused on individuals with DKD. However, it’s essential to emphasize that the FIDELIO-DKD and FIGARO-DKD trials specifically enrolled participants with DKD, characterized by an eGFR of ≥25 mL/min/1.73 m^2^, normal serum potassium levels, and albuminuria. Consequently, the current approval of FIN cannot be generalized to the entire population of individuals with DKD. Hence, further research is needed to clarify this aspect. Ongoing studies are exploring the role of triple therapy, consisting of RAS blockade, SGLT2i, and FIN, in individuals with DKD (CONFIDENCE study, NCT05254002) (128). Additionally, there are investigations into the efficacy and safety of FIN in subjects with CKD who do not have diabetes (FIND-CKD study, NCT05047263) (129), as well as an examination of treatment patterns in patients with DKD treated with FIN in routine clinical practice, including safety assessments (FINE-REAL study, NCT05348733) (130).

The CONFIDENCE trial is an ongoing phase II randomized controlled trial designed to investigate the combination of FIN and empagliflozin compared with each drug alone in 807 participants with T2D, stage 2–3 CKD and UACR ranging from ≥300 to <5000 mg/g (128). This trial is estimated to be completed by 2024 and will comprehensively evaluate the cumulative efficacy, safety, and tolerability of dual therapy (128). The study will primarily focus on endpoints related to UACR, change in eGFR, and the incidence of hyperkalemia (128). However, as of this writing, no prospective clinical trials were planned to judge the combination of FIN and GLP-1RAs in patients with DKD.

Initiated in September 2021, the FIND-CKD trial includes non-diabetic CKD patients with an eGFR ranging from 25–90 mL/min/1.73 m^2^ and a UACR between ≥200 to ≤3500 mg/gCr (129). The primary objective of this study is to assess the change in eGFR from baseline to 32 months in both the placebo and FIN groups.

The ongoing FINE-REAL study (130) aims to demonstrate treatment patterns in patients with DKD receiving FIN in routine clinical practice and assess the safety of FIN. FINE-REAL will provide meaningful insights into DKD patients treated with FIN, capturing AEs, specifically hyperkalemia, and identifying how they are handled in routine clinical care. The FINE-REAL study will aid to inform decision-making about initiating FIN in individuals with DKD and also shed light on the dynamics of adoption of new therapies in different regions and health systems, providing conducive perspectives for international guidance and implementation.

## 9 Conclusion and future advancement

FIN is a third-generation, selective and potent NS-MRA that has been illustrated in clinical trials to slow CKD progression, reduce the risk of CV events development and hyperkalemia compared to traditional steroidal MRAs in patients with DKD through specific impacts on inflammatory and fibrotic pathways. As such, FIN is a valuable addition to the treatment landscape for managing DKD. However, more clinical trials and deep mechanism research are needed to provide conclusive evidence for the combination treatment of FIN with ACEI/ARB and SGLT2i/GLP-1RAs. Additionally, further large-scale investigations are supposed to confirm the efficacy and safety of FIN in DKD with an eGFR below 25 mL/min/1.73 m^2^ and assess the clinical usage of FIN in patients with CKD but without diabetes. Besides, few studies have assessed whether this novel NS-MRA retain their beneficial effects across various kidney diseases as those observed with extant steroidal MRA, and there is no evidence for use in other settings like resistant hypertension, ascites due to cirrhosis, or primary hyperaldosteronism as compared to the first- and second-generation MRAs spironolactone or eplerenone. In addition, whether MR blockade alleviates IR injury in kidney transplantation is an intriguing topic. To justify these usages, comparative studies will need to be conducted for these specific conditions.

## Author contributions

WC: Investigation, Methodology, Visualization, Writing – original draft. LZ: Investigation, Methodology, Validation, Writing – review & editing. JW: Investigation, Methodology, Visualization, Writing – review & editing. YL: Investigation, Methodology, Supervision, Visualization, Writing – review & editing. TZ: Investigation, Methodology, Project administration, Supervision, Validation, Visualization, Writing – review & editing.

## Glossary

**Table:**

DKD	diabetic kidney disease
CKD	chronic kidney disease
T2D	type 2 diabetes
CV	cardiovascular
MRs	mineralocorticoid receptors
MRAs	MR antagonists
FIN	finerenone
CVD	CV disease
eGFR	estimated glomerular filtration rate
AKI	acute kidney injury
ESKD	end-stage kidney disease
BP	blood pressure
RAS	renin-angiotensin system
ACEi	angiotensin-converting enzyme inhibitors
ARB	angiotensin receptor blockers
SGLT2i	sodium-glucose co-transporters-2 inhibitors
NS-MRAs	nonsteroidal MRAs
DOCA	deoxycorticosterone acetate
IR	ischemia-reperfusion
MWF	munich Wistar frömter
HS	high salt
UACR	urine albumin-creatinine ratio
ARTS-DN	Mineralocorticoid Receptor Antagonist Tolerability Study–Diabetic Nephropathy
FIDELIO-DKD	Finerenone in Reducing Kidney Failure and Disease Progression in Diabetic Kidney Disease
FIGARO-DKD	Finerenone in Reducing Cardiovascular Mortality and Morbidity in Diabetic Kidney Disease
RCT	randomized controlled trial
MI	myocardial infarction
HF	heart failure
FDA	Food and Drug Administration
EMA	European Medicines Agency
ADA	American Diabetes Association
AACE	American Association of Clinical Endocrinology
KDIGO	Kidney Disease: Improving Global Outcomes
ARTS	Mineralocorticoid Receptor Antagonist Tolerability Study
HFrEF	heart failure with reduced ejection fraction
pro-BNP	pro-B-type natriuretic peptide
WRF	worsening renal function
ARTS-HF	ARTS-Heart Failure
HFpEF	heart failure with preserved ejection fraction
AEs	adverse effects
TEAEs	treatment-emergent AEs
GLP-1RAs	glucagon-like peptide-1 receptor agonist
TGF-β1	transforming growth factor-β-1
PAI-1	plasminogen activator inhibitor-1
Rac1	Ras-related C3 botulinum toxin substrate 1
NOX	NADPH oxidases
ROS	reactive oxygen species
NO	Nitric oxide
SMCs	smooth muscle cells
KIM-1	kidney injury molecule 1
NGAL	neutrophil gelatinase-associated lipocalin
TNF-α	tumor necrosis factor-α
MMP	matrix metalloproteinase
CCL-2	C-C motif chemokine ligand 2
IL	interleukin
ROR	retinoid-related orphan receptor
MCP-1	monocyte chemoattractant protein-1
OPN	osteopontin
COL1A1	collagen type I α 1 chain
NKD2	naked cuticle homolog 2
VSMC	vascular smooth muscle cell
SOD	superoxide dismutase
ENaC	epithelial sodium channel
NCC	thiazide-sensitive sodium chloride cotransporter
UNX	Uninephrectomized

## References

[1] Romagnani P Remuzzi G Glassock R Levin A Jager KJ Tonelli M. Chronic kidney disease. Nat Rev Dis Primers (2017) 3:17088. doi: 10.1038/nrdp.2017.88 29168475

[2] Levey AS de Jong PE Coresh J El Nahas M Astor BC Matsushita K. The definition, classification, and prognosis of chronic kidney disease: a KDIGO Controversies Conference report. Kidney Int (2011) 80(1):17–28. doi: 10.1038/ki.2010.483 21150873

[3] GBD Chronic Kidney Disease Collaboration. Global, regional, and national burden of chronic kidney disease, 1990-2017: a systematic analysis for the Global Burden of Disease Study 2017. Lancet (2020) 395(10225):709–33. doi: 10.1016/S0140-6736(19)32977-0 PMC7049905 32061315

[4] Ortiz A Asociación Información Enfermedades Renales Genéticas (AIRG-E) European Kidney Patients’ Federation (EKPF) Federación Nacional de Asociaciones para la Lucha Contra las Enfermedades del Riñón (ALCER) Fundación Renal Íñigo Álvarez de Toledo (FRIAT) Red de Investigación Renal (REDINREN). RICORS2040: the need for collaborative research in chronic kidney disease. Clin Kidney J (2022) 15(3):372–87. doi: 10.1093/ckj/sfab170 PMC8862113 35211298

[5] Jager KJ Kovesdy C Langham R Rosenberg M Jha V Zoccali C. A single number for advocacy and communication-worldwide more than 850 million individuals have kidney diseases. Nephrol Dial Transpl (2019) 34(11):1803–5. doi: 10.1093/ndt/gfz174 31566230

[6] Gansevoort RT Correa-Rotter R Hemmelgarn BR Jafar TH Heerspink HJL Mann JF. Chronic kidney disease and cardiovascular risk: epidemiology, mechanisms, and prevention. Lancet (2013) 382(9889):339–52. doi: 10.1016/S0140-6736(13)60595-4 23727170

[7] Doshi SM Friedman AN. Diagnosis and management of type 2 diabetic kidney disease. Clin J Am Soc Nephrol (2017) 12(8):1366–73. doi: 10.2215/CJN.11111016 PMC5544517 28280116

[8] Li H Lu W Wang A Jiang H Lyu J. Changing epidemiology of chronic kidney disease as a result of type 2 diabetes mellitus from 1990 to 2017: Estimates from Global Burden of Disease 2017. J Diabetes Investig (2021) 12(3):346–56. doi: 10.1111/jdi.13355 PMC7926234 32654341

[9] Alicic RZ Rooney MT Tuttle KR. Diabetic kidney disease: challenges, progress, and possibilities. Clin J Am Soc Nephrol (2017) 12(12):2032–45. doi: 10.2215/CJN.11491116 PMC5718284 28522654

[10] Rangaswami J Bhalla V de Boer IH Staruschenko A Sharp JA Singh RR. Cardiorenal protection with the newer antidiabetic agents in patients with diabetes and chronic kidney disease: A scientific statement from the American heart association. Circulation (2020) 142(17):e265–86. doi: 10.1161/CIR.0000000000000920 32981345

[11] Narres M Claessen H Droste S Kvitkina T Koch M Kuss O. The incidence of end-stage renal disease in the diabetic (Compared to the non-diabetic) population: A systematic review. PloS One (2016) 11(1):e0147329. doi: 10.1371/journal.pone.0147329 26812415 PMC4727808

[12] Nichols GA Déruaz-Luyet A Hauske SJ Brodovicz KG. The association between estimated glomerular filtration rate, albuminuria, and risk of cardiovascular hospitalizations and all-cause mortality among patients with type 2 diabetes. J Diabetes Complications (2018) 32(3):291–7. doi: 10.1016/j.jdiacomp.2017.12.003 29352693

[13] Chaudhuri A Ghanim H Arora P. Improving the residual risk of renal and cardiovascular outcomes in diabetic kidney disease: A review of pathophysiology, mechanisms, and evidence from recent trials. Diabetes Obes Metab (2022) 24(3):365–76. doi: 10.1111/dom.14601 PMC9300158 34779091

[14] Wen CP Chang CH Tsai MK Lee JH Lu PJ Tsai SP. Diabetes with early kidney involvement may shorten life expectancy by 16 years. Kidney Int (2017) 92(2):388–96. doi: 10.1016/j.kint.2017.01.030 28577854

[15] Afkarian M Sachs MC Kestenbaum B Hirsch IB Tuttle KR Himmelfarb J. Kidney disease and increased mortality risk in type 2 diabetes. J Am Soc Nephrol (2013) 24(2):302–8. doi: 10.1681/ASN.2012070718 PMC3559486 23362314

[16] Zoccali C Vanholder R Massy ZA Ortiz A Sarafidis P Dekker FW. The systemic nature of CKD. Nat Rev Nephrol (2017) 13(6):344–58. doi: 10.1038/nrneph.2017.52 28435157

[17] Bertocchio JP Warnock DG Jaisser F. Mineralocorticoid receptor activation and blockade: an emerging paradigm in chronic kidney disease. Kidney Int (2011) 79(10):1051–60. doi: 10.1038/ki.2011.48 21412221

[18] Shibata S Nagase M Yoshida S Kawarazaki W Kurihara H Tanaka H. Modification of mineralocorticoid receptor function by Rac1 GTPase: implication in proteinuric kidney disease. Nat Med (2008) 14(12):1370–6. doi: 10.1038/nm.1879 19029984

[19] Quinkler M Zehnder D Eardley KS Lepenies J Howie AJ Hughes SV. Increased expression of mineralocorticoid effector mechanisms in kidney biopsies of patients with heavy proteinuria. Circulation (2005) 112(10):1435–43. doi: 10.1161/CIRCULATIONAHA.105.539122 16145013

[20] Bădilă E. The expanding class of mineralocorticoid receptor modulators: New ligands for kidney, cardiac, vascular, systemic and behavioral selective actions. Acta Endocrinol (Buchar) (2020) 16(4):487–96. doi: 10.4183/aeb.2020.487 PMC8126399 34084241

[21] Kawarazaki W Nagase M Yoshida S Takeuchi M Ishizawa K Ayuzawa N. Angiotensin II- and salt-induced kidney injury through Rac1-mediated mineralocorticoid receptor activation. J Am Soc Nephrol (2012) 23(6):997–1007. doi: 10.1681/ASN.2011070734 22440899 PMC3358757

[22] Kawarazaki W Fujita T. The role of aldosterone in obesity-related hypertension. Am J Hypertens (2016) 29(4):415–23. doi: 10.1093/ajh/hpw003 PMC4886496 26927805

[23] Jaques DA Wuerzner G Ponte B. Sodium intake as a cardiovascular risk factor: A narrative review. Nutrients (2021) 13(9):3177. doi: 10.3390/nu13093177 34579054 PMC8470268

[24] Shibata S Mu S Kawarazaki H Muraoka K Ishizawa K Yoshida S. Rac1 GTPase in rodent kidneys is essential for salt-sensitive hypertension via a mineralocorticoid receptor-dependent pathway. J Clin Invest (2011) 121(8):3233–43. doi: 10.1172/JCI43124 PMC3148723 21765214

[25] Epstein M. Aldosterone and mineralocorticoid receptor signaling as determinants of cardiovascular and renal injury: from Hans Selye to the present. Am J Nephrol (2021) 52(3):209–16. doi: 10.1159/000515622 33857953

[26] Barrera-Chimal J Girerd S Jaisser F. Mineralocorticoid receptor antagonists and kidney diseases: pathophysiological basis. Kidney Int (2019) 96(2):302–19. doi: 10.1016/j.kint.2019.02.030 31133455

[27] Barrera-Chimal J Estrela GR Lechner SM Giraud S El Moghrabi S Kaaki S. The myeloid mineralocorticoid receptor controls inflammatory and fibrotic responses after renal injury via macrophage interleukin-4 receptor signaling. Kidney Int (2018) 93(6):1344–55. doi: 10.1016/j.kint.2017.12.016 29548765

[28] Erraez S López-Mesa M Gómez-Fernández P. Mineralcorticoid receptor blockers in chronic kidney disease. Nefrologia (Engl Ed) (2021) 41(3):258–75. doi: 10.1016/j.nefroe.2021.08.001 36166243

[29] Huang LL Nikolic-Paterson DJ Han Y Ozols E Ma FY Young MJ. Myeloid mineralocorticoid receptor activation contributes to progressive kidney disease. J Am Soc Nephrol (2014) 25(10):2231–40. doi: 10.1681/ASN.2012111094 PMC4178428 24700867

[30] Brown NJ. Contribution of aldosterone to cardiovascular and renal inflammation and fibrosis. Nat Rev Nephrol (2013) 9(8):459–69. doi: 10.1038/nrneph.2013.110 PMC3922409 23774812

[31] Remuzzi G Cattaneo D Perico N. The aggravating mechanisms of aldosterone on kidney fibrosis. J Am Soc Nephrol (2008) 19(8):1459–62. doi: 10.1681/ASN.2007101079 18550649

[32] Barrera-Chimal J Lima-Posada I Bakris GL Jaisser F. Mineralocorticoid receptor antagonists in diabetic kidney disease - mechanistic and therapeutic effects. Nat Rev Nephrol (2022) 18(1):56–70. doi: 10.1038/s41581-021-00490-8 34675379

[33] Tesch GH Young MJ. Mineralocorticoid receptor signaling as a therapeutic target for renal and cardiac fibrosis. Front Pharmacol (2017) 8:313. doi: 10.3389/fphar.2017.00313 28611666 PMC5447060

[34] Handelsman Y Butler J Bakris GL DeFronzo RA Fonarow GC Green JB. Early intervention and intensive management of patients with diabetes, cardiorenal, and metabolic diseases. J Diabetes Complications (2023) 37(2):108389. doi: 10.1016/j.jdiacomp.2022.108389 36669322

[35] Brenner BM Cooper ME de Zeeuw D Keane WF Mitch WE Parving HH. Effects of losartan on renal and cardiovascular outcomes in patients with type 2 diabetes and nephropathy. N Engl J Med (2001) 345(12):861–9. doi: 10.1056/NEJMoa011161 11565518

[36] Lewis EJ Hunsicker LG Clarke WR Berl T Pohl MA Lewis JB. Renoprotective effect of the angiotensin-receptor antagonist irbesartan in patients with nephropathy due to type 2 diabetes. N Engl J Med (2001) 345(12):851–60. doi: 10.1056/NEJMoa011303 11565517

[37] Sarafidis P Ferro CJ Morales E Ortiz A Malyszko J Hojs R. SGLT-2 inhibitors and GLP-1 receptor agonists for nephroprotection and cardioprotection in patients with diabetes mellitus and chronic kidney disease. A consensus statement by the EURECA-m and the DIABESITY working groups of the ERA-EDTA. Nephrol Dial Transpl (2019) 34(2):208–30. doi: 10.1093/ndt/gfy407 30753708

[38] Perkovic V Jardine MJ Neal B Bompoint S Heerspink HJL Charytan DM. Canagliflozin and renal outcomes in type 2 diabetes and nephropathy. N Engl J Med (2019) 380(24):2295–306. doi: 10.1056/NEJMoa1811744 30990260

[39] Heerspink HJL Stefánsson BV Correa-Rotter R Chertow GM Greene T Hou FF. Dapagliflozin in patients with chronic kidney disease. N Engl J Med (2020) 383(15):1436–46. doi: 10.1056/NEJMoa2024816 32970396

[40] Lewis EJ Hunsicker LG Bain RP Rohde RD. The effect of angiotensin-converting-enzyme inhibition on diabetic nephropathy. The Collaborative Study Group. N Engl J Med (1993) 329(20):1456–62. doi: 10.1056/NEJM199311113292004 8413456

[41] Terpening CM. Prevention of cardiovascular events in patients with chronic kidney disease. Ann Pharmacother (2023) 57(12):1425–35. doi: 10.1177/10600280231165774 37029538

[42] D’Marco L PuChades MJ Gandía L Forquet C Giménez-Civera E Panizo N. Finerenone: A potential treatment for patients with chronic kidney disease and type 2 diabetes mellitus. touchREV Endocrinol (2021) 17(2):84–7. doi: 10.17925/EE.2021.17.2.84 PMC8676102 35118452

[43] DuPont JJ Jaffe IZ. 30 YEARS OF THE MINERALOCORTICOID RECEPTOR: The role of the mineralocorticoid receptor in the vasculature. J Endocrinol (2017) 234(1):T67–82. doi: 10.1530/JOE-17-0009 PMC5518626 28634267

[44] Alicic RZ Johnson EJ Tuttle KR. Inflammatory mechanisms as new biomarkers and therapeutic targets for diabetic kidney disease. Adv Chronic Kidney Dis (2018) 25(2):181–91. doi: 10.1053/j.ackd.2017.12.002 29580582

[45] González-Blázquez R Somoza B Gil-Ortega M Martín Ramos M Ramiro-Cortijo D Vega-Martín E. Finerenone attenuates endothelial dysfunction and albuminuria in a chronic kidney disease model by a reduction in oxidative stress. Front Pharmacol (2018) 9:1131. doi: 10.3389/fphar.2018.01131 30356804 PMC6189469

[46] Gil-Ortega M Vega-Martín E Martín-Ramos M González-Blázquez R Pulido-Olmo H Ruiz-Hurtado G. Finerenone reduces intrinsic arterial stiffness in Munich Wistar Frömter rats, a genetic model of chronic kidney disease. Am J Nephrol (2020) 51(4):294–303. doi: 10.1159/000506275 32088716

[47] Dutzmann J Musmann RJ Haertlé M Daniel JM Sonnenschein K Schäfer A. The novel mineralocorticoid receptor antagonist finerenone attenuates neointima formation after vascular injury. PLoS One (2017) 12(9):e0184888. doi: 10.1371/journal.pone.0184888 28926607 PMC5605005

[48] Ng KP Arnold J Sharif A Gill P Townend JN Ferro CJ. Cardiovascular actions of mineralocorticoid receptor antagonists in patients with chronic kidney disease: A systematic review and meta-analysis of randomized trials. J Renin Angiotensin Aldosterone Syst (2015) 16(3):599–613. doi: 10.1177/1470320315575849 25784710

[49] Currie G Taylor AHM Fujita T Ohtsu H Lindhardt M Rossing P. Effect of mineralocorticoid receptor antagonists on proteinuria and progression of chronic kidney disease: a systematic review and meta-analysis. BMC Nephrol (2016) 17(1):127. doi: 10.1186/s12882-016-0337-0 27609359 PMC5015203

[50] Sarafidis PA Memmos E Alexandrou ME Papagianni A. Mineralocorticoid receptor antagonists for nephroprotection: current evidence and future perspectives. Curr Pharm Des (2018) 24(46):5528–36. doi: 10.2174/1381612825666190306162658 30848187

[51] Ai Dhaybi O Bakris GL. Renal targeted therapies of antihypertensive and cardiovascular drugs for patients with stages 3 through 5d kidney disease. Clin Pharmacol Ther (2017) 102(3):450–8. doi: 10.1002/cpt.758 28589612

[52] Pitt B Rossignol P. The safety of mineralocorticoid receptor antagonists (MRAs) in patients with heart failure. Expert Opin Drug Saf (2016) 15(5):659–65. doi: 10.1517/14740338.2016.1163335 26958701

[53] Bolignano D Palmer SC Navaneethan SD Strippoli GFM. Aldosterone antagonists for preventing the progression of chronic kidney disease. Cochrane Database Syst Rev (2014) 4:CD007004. doi: 10.1002/14651858.CD007004.pub3 24782282

[54] Trevisan M de Deco P Xu H Evans M Lindholm B Bellocco R. Incidence, predictors and clinical management of hyperkalaemia in new users of mineralocorticoid receptor antagonists. Eur J Heart Fail (2018) 20(8):1217–26. doi: 10.1002/ejhf.1199 PMC6607478 29667759

[55] Kolkhof P Bärfacker L. 30 YEARS OF THE MINERALOCORTICOID RECEPTOR: Mineralocorticoid receptor antagonists: 60 years of research and development. J Endocrinol (2017) 234(1):T125–40. doi: 10.1530/JOE-16-0600 PMC5488394 28634268

[56] Lazich I Bakris GL. Prediction and management of hyperkalemia across the spectrum of chronic kidney disease. Semin Nephrol (2014) 34(3):333–9. doi: 10.1016/j.semnephrol.2014.04.008 25016403

[57] Kolkhof P Joseph A Kintscher U. Nonsteroidal mineralocorticoid receptor antagonism for cardiovascular and renal disorders - New perspectives for combination therapy. Pharmacol Res (2021) 172:105859. doi: 10.1016/j.phrs.2021.105859 34461222

[58] Bärfacker L Kuhl A Hillisch A Grosser R Figueroa-Pérez S Heckroth H. Discovery of BAY 94-8862: a nonsteroidal antagonist of the mineralocorticoid receptor for the treatment of cardiorenal diseases. ChemMedChem (2012) 7(8):1385–403. doi: 10.1002/cmdc.201200081 22791416

[59] Kintscher U Bakris GL Kolkhof P. Novel non-steroidal mineralocorticoid receptor antagonists in cardiorenal disease. Br J Pharmacol (2022) 179(13):3220–34. doi: 10.1111/bph.15747 34811750

[60] Lorente-Ros M Aguilar-Gallardo JS Shah A Narasimhan B Aronow WS. An overview of mineralocorticoid receptor antagonists as a treatment option for patients with heart failure: the current state-of-the-art and future outlook. Expert Opin Pharmacother (2022) 23(15):1737–51. doi: 10.1080/14656566.2022.2138744 36262014

[61] Heinig R Kimmeskamp-Kirschbaum N Halabi A Lentini S. Pharmacokinetics of the novel nonsteroidal mineralocorticoid receptor antagonist finerenone (BAY 94-8862) in individuals with renal impairment. Clin Pharmacol Drug Dev (2016) 5(6):488–501. doi: 10.1002/cpdd.263 27431783

[62] Shibata S. 30 YEARS OF THE MINERALOCORTICOID RECEPTOR: Mineralocorticoid receptor and NaCl transport mechanisms in the renal distal nephron. J Endocrinol (2017) 234(1):T35–47. doi: 10.1530/JOE-16-0669 28341694

[63] Lother A Jaisser F Wenzel UO. Emerging fields for therapeutic targeting of the aldosterone-mineralocorticoid receptor signaling pathway. Br J Pharmacol (2022) 179(13):3099–102. doi: 10.1111/bph.15808 35174485

[64] Verma A Vaidya A Subudhi S Waikar SS. Aldosterone in chronic kidney disease and renal outcomes. Eur Heart J (2022) 43(38):3781–91. doi: 10.1093/eurheartj/ehac352 PMC10147385 36219773

[65] Bauersachs J Jaisser F Toto R. Mineralocorticoid receptor activation and mineralocorticoid receptor antagonist treatment in cardiac and renal diseases. Hypertension (2015) 65(2):257–63. doi: 10.1161/HYPERTENSIONAHA.114.04488 25368026

[66] Jaisser F Farman N. Emerging roles of the mineralocorticoid receptor in pathology: toward new paradigms in clinical pharmacology. Pharmacol Rev (2016) 68(1):49–75. doi: 10.1124/pr.115.011106 26668301

[67] Iyer A Chan V Brown L. The DOCA-salt hypertensive rat as a model of cardiovascular oxidative and inflammatory stress. Curr Cardiol Rev (2010) 6(4):291–7. doi: 10.2174/157340310793566109 PMC3083810 22043205

[68] Araujo M Wilcox CS. Oxidative stress in hypertension: role of the kidney. Antioxid Redox Signal (2014) 20(1):74–101. doi: 10.1089/ars.2013.5259 23472618 PMC3880923

[69] Nishiyama A Yao L Nagai Y Miyata K Yoshizumi M Kagami S. Possible contributions of reactive oxygen species and mitogen-activated protein kinase to renal injury in aldosterone/salt-induced hypertensive rats. Hypertension (2004) 43(4):841–8. doi: 10.1161/01.HYP.0000118519.66430.22 14769808

[70] Barrera-Chimal J Prince S Fadel F El Moghrabi S Warnock DG Kolkhof P. Sulfenic acid modification of endothelin B receptor is responsible for the benefit of a nonsteroidal mineralocorticoid receptor antagonist in renal ischemia. J Am Soc Nephrol (2016) 27(2):398–404. doi: 10.1681/ASN.2014121216 26361797 PMC4731121

[71] Barrera-Chimal J André-Grégoire G Nguyen Dinh Cat A Lechner SM Cau J Prince S. Benefit of mineralocorticoid receptor antagonism in AKI: role of vascular smooth muscle Rac1. J Am Soc Nephrol (2017) 28(4):1216–26. doi: 10.1681/ASN.2016040477 PMC5373452 28087726

[72] Lattenist L Lechner SM Messaoudi S Le Mercier A El Moghrabi S Prince S. Nonsteroidal mineralocorticoid receptor antagonist finerenone protects against acute kidney injury-mediated chronic kidney disease: role of oxidative stress. Hypertension (2017) 69(5):870–8. doi: 10.1161/HYPERTENSIONAHA.116.08526 28320854

[73] Rossing P Anker SD Filippatos G Pitt B Ruilope LM Birkenfeld AL. Finerenone in patients with chronic kidney disease and type 2 diabetes by sodium-glucose cotransporter 2 inhibitor treatment: the FIDELITY analysis. Diabetes Care (2022) 45(12):2991–8. doi: 10.2337/dc22-0294 PMC9862372 35972218

[74] Huen SC Cantley LG. Macrophage-mediated injury and repair after ischemic kidney injury. Pediatr Nephrol (2015) 30(2):199–209. doi: 10.1007/s00467-013-2726-y 24442822 PMC5048744

[75] van der Heijden CDCC Deinum J Joosten LAB Netea MG Riksen NP. The mineralocorticoid receptor as a modulator of innate immunity and atherosclerosis. Cardiovasc Res (2018) 114(7):944–53. doi: 10.1093/cvr/cvy092 29668907

[76] Luettges K Bode M Diemer JN Schwanbeck J Wirth EK Klopfleisch R. Finerenone reduces renal RORγt γδ T cells and protects against cardiorenal damage. Am J Nephrol (2022) 53(7):552–64. doi: 10.1159/000524940 35675794

[77] Kolkhof P Delbeck M Kretschmer A Steinke W Hartmann E Bärfacker L. Finerenone, a novel selective nonsteroidal mineralocorticoid receptor antagonist protects from rat cardiorenal injury. J Cardiovasc Pharmacol (2014) 64(1):69–78. doi: 10.1097/FJC.0000000000000091 24621652

[78] Murea M Register TC Divers J Bowden DW Carr JJ Hightower CR. Relationships between serum MCP-1 and subclinical kidney disease: African American-Diabetes Heart Study. BMC Nephrol (2012) 13:148. doi: 10.1186/1471-2369-13-148 23151275 PMC3534523

[79] Bolignano D Lacquaniti A Coppolino G Donato V Campo S Fazio MR. Neutrophil gelatinase-associated lipocalin (NGAL) and progression of chronic kidney disease. Clin J Am Soc Nephrol (2009) 4(2):337–44. doi: 10.2215/CJN.03530708 PMC2637601 19176795

[80] Steinbrenner I Sekula P Kotsis F von Cube M Cheng Y Nadal J. Association of osteopontin with kidney function and kidney failure in chronic kidney disease patients: the GCKD study. Nephrol Dial Transpl (2023) 38(6):1430–8. doi: 10.1093/ndt/gfac173 35524694

[81] Hirohama D Nishimoto M Ayuzawa N Kawarazaki W Fujii W Oba S. Activation of rac1-mineralocorticoid receptor pathway contributes to renal injury in salt-loaded db/db mice. Hypertension (2021) 78(1):82–93. doi: 10.1161/HYPERTENSIONAHA.121.17263 34058848

[82] Mansour SG Puthumana J Coca SG Gentry M Parikh CR. Biomarkers for the detection of renal fibrosis and prediction of renal outcomes: a systematic review. BMC Nephrol (2017) 18(1):72. doi: 10.1186/s12882-017-0490-0 28219345 PMC5319065

[83] Kolkhof P Hartmann E Freyberger A Pavkovic M Mathar I Sandner P. Effects of finerenone combined with empagliflozin in a model of hypertension-induced end-organ damage. Am J Nephrol (2021) 52(8):642–52. doi: 10.1159/000516213 PMC8619789 34111864

[84] Droebner K Pavkovic M Grundmann M Hartmann E Goea L Nordlohne J. Direct blood pressure-independent anti-fibrotic effects by the selective nonsteroidal mineralocorticoid receptor antagonist finerenone in progressive models of kidney fibrosis. Am J Nephrol (2021) 52(7):588–601. doi: 10.1159/000518254 34515038

[85] Kuppe C Ibrahim MM Kranz J Zhang X Ziegler S Perales-Patón J. Decoding myofibroblast origins in human kidney fibrosis. Nature (2021) 589(7841):281–6. doi: 10.1038/s41586-020-2941-1 PMC7611626 33176333

[86] Lima-Posada I Stephan Y Soulié M Palacios-Ramirez R Bonnard B Nicol L. Benefits of the non-steroidal mineralocorticoid receptor antagonist finerenone in metabolic syndrome-related heart failure with preserved ejection fraction. Int J Mol Sci (2023) 24(3):2536. doi: 10.3390/ijms24032536 36768859 PMC9916671

[87] Pitt B Filippatos G Agarwal R Anker SD Bakris GL Rossing P. Cardiovascular events with finerenone in kidney disease and type 2 diabetes. N Engl J Med (2021) 385(24):2252–63. doi: 10.1056/NEJMoa2110956 34449181

[88] Bakris GL Agarwal R Anker SD Pitt B Ruilope LM Rossing P. Effect of finerenone on chronic kidney disease outcomes in type 2 diabetes. N Engl J Med (2020) 383(23):2219–29. doi: 10.1056/NEJMoa2025845 33264825

[89] Pitt B Kober L Ponikowski P Gheorghiade M Filippatos G Krum H. Safety and tolerability of the novel non-steroidal mineralocorticoid receptor antagonist BAY 94-8862 in patients with chronic heart failure and mild or moderate chronic kidney disease: a randomized, double-blind trial. Eur Heart J (2013) 34(31):2453–63. doi: 10.1093/eurheartj/eht187 PMC3743070 23713082

[90] Filippatos G Anker SD Böhm M Gheorghiade M Køber L Krum H. A randomized controlled study of finerenone vs. eplerenone in patients with worsening chronic heart failure and diabetes mellitus and/or chronic kidney disease. Eur Heart J (2016) 37(27):2105–14. doi: 10.1093/eurheartj/ehw132 PMC4946749 27130705

[91] Bakris GL Agarwal R Chan JC Cooper ME Gansevoort RT Haller H. Effect of finerenone on albuminuria in patients with diabetic nephropathy: A randomized clinical trial. JAMA (2015) 314(9):884–94. doi: 10.1001/jama.2015.10081 26325557

[92] Katayama S Yamada D Nakayama M Yamada T Myoishi M Kato M. A randomized controlled study of finerenone versus placebo in Japanese patients with type 2 diabetes mellitus and diabetic nephropathy. J Diabetes Complications (2017) 31(4):758–65. doi: 10.1016/j.jdiacomp.2016.11.021 28025025

[93] Snelder N Heinig R Drenth HJ Joseph A Kolkhof P Lippert J. Population pharmacokinetic and exposure-response analysis of finerenone: insights based on phase IIb data and simulations to support dose selection for pivotal trials in type 2 diabetes with chronic kidney disease. Clin Pharmacokinet (2020) 59(3):359–70. doi: 10.1007/s40262-019-00820-x PMC7051931 31583611

[94] Agarwal R Filippatos G Pitt B Anker SD Rossing P Joseph A. Cardiovascular and kidney outcomes with finerenone in patients with type 2 diabetes and chronic kidney disease: the FIDELITY pooled analysis. Eur Heart J (2022) 43(6):474–84. doi: 10.1093/eurheartj/ehab777 PMC8830527 35023547

[95] Goulooze SC Heerspink HJL van Noort M Snelder N Brinker M Lippert J. Dose-exposure-response analysis of the nonsteroidal mineralocorticoid receptor antagonist finerenone on UACR and eGFR: an analysis from FIDELIO-DKD. Clin Pharmacokinet (2022) 61(7):1013–25. doi: 10.1007/s40262-022-01124-3 PMC9287422 35508594

[96] Filippatos G Bakris GL Pitt B Agarwal R Rossing P Ruilope LM. Finerenone reduces new-onset atrial fibrillation in patients with chronic kidney disease and type 2 diabetes. J Am Coll Cardiol (2021) 78(2):142–52. doi: 10.1016/j.jacc.2021.04.079 34015478

[97] Mende CW Samarakoon R Higgins PJ. Mineralocorticoid receptor-associated mechanisms in diabetic kidney disease and clinical significance of mineralocorticoid receptor antagonists. Am J Nephrol (2023) 54(1–2):50–61. doi: 10.1159/000528783 36682353 PMC10273909

[98] Ruilope LM Pitt B Anker SD Rossing P Kovesdy CP Pecoits-Filho R. Kidney outcomes with finerenone: an analysis from the FIGARO-DKD study. Nephrol Dial Transpl (2023) 38(2):372–83. doi: 10.1093/ndt/gfac157 PMC9923706 35451488

[99] Agarwal R Pitt B Palmer BF Kovesdy CP Burgess E Filippatos G. A comparative *post hoc* analysis of finerenone and spironolactone in resistant hypertension in moderate-to-advanced chronic kidney disease. Clin Kidney J (2023) 16(2):293–302. doi: 10.1093/ckj/sfac234 36864892 PMC9972517

[100] Bakris GL Ruilope LM Anker SD Filippatos G Pitt B Rossing P. A prespecified exploratory analysis from FIDELITY examined finerenone use and kidney outcomes in patients with chronic kidney disease and type 2 diabetes. Kidney Int (2023) 103(1):196–206. doi: 10.1016/j.kint.2022.08.040 36367466

[101] Sarafidis P Agarwal R Pitt B Wanner C Filippatos G Boletis J. Outcomes with finerenone in participants with stage 4 CKD and type 2 diabetes: A FIDELITY subgroup analysis. Clin J Am Soc Nephrol (2023) 18(5):602–12. doi: 10.2215/CJN.0000000000000149 PMC10278789 36927680

[102] Mima A Lee R Murakami A Gotoda H Akai R Kidooka S. Effect of finerenone on diabetic kidney disease outcomes with estimated glomerular filtration rate below 25 mL/min/1. 73 m2. Metabol Open (2023) 19:100251. doi: 10.1016/j.metop.2023.100251 37497038 PMC10366575

[103] Filippatos G Anker SD August P Coats AJS Januzzi JL Mankovsky B. Finerenone and effects on mortality in chronic kidney disease and type 2 diabetes: a FIDELITY analysis. Eur Heart J Cardiovasc Pharmacother (2023) 9(2):183–91. doi: 10.1093/ehjcvp/pvad001 PMC9892867 36639130

[104] McGill JB Agarwal R Anker SD Bakris GL Filippatos G Pitt B. Effects of finerenone in people with chronic kidney disease and type 2 diabetes are independent of HbA1c at baseline, HbA1c variability, diabetes duration and insulin use at baseline. Diabetes Obes Metab (2023) 25(6):1512–22. doi: 10.1111/dom.14999 36722675

[105] Rossing P Burgess E Agarwal R Anker SD Filippatos G Pitt B. Finerenone in patients with chronic kidney disease and type 2 diabetes according to baseline HbA1c and insulin use: an analysis from the FIDELIO-DKD study. Diabetes Care (2022) 45(4):888–97. doi: 10.2337/dc21-1944 PMC9271031 35061867

[106] Filippatos G Pitt B Agarwal R Farmakis D Ruilope LM Rossing P. Finerenone in patients with chronic kidney disease and type 2 diabetes with and without heart failure: a prespecified subgroup analysis of the FIDELIO-DKD trial. Eur J Heart Fail (2022) 24(6):996–1005. doi: 10.1002/ejhf.2469 35239204 PMC9541504

[107] Filippatos G Anker SD Agarwal R Ruilope LM Rossing P Bakris GL. Finerenone reduces risk of incident heart failure in patients with chronic kidney disease and type 2 diabetes: analyses from the FIGARO-DKD trial. Circulation (2022) 145(6):437–47. doi: 10.1161/CIRCULATIONAHA.121.057983 PMC8812430 34775784

[108] Filippatos G Anker SD Pitt B McGuire DK Rossing P Ruilope LM. Finerenone efficacy in patients with chronic kidney disease, type 2 diabetes and atherosclerotic cardiovascular disease. Eur Heart J Cardiovasc Pharmacother (2022) 9(1):85–93. doi: 10.1093/ehjcvp/pvac054 36251465 PMC9753093

[109] Finerenone (Kerendia) for chronic kidney disease. Med Lett Drugs Ther (2021) 63(1631):131–2. 34544101

[110] Vizcaya D Kovesdy CP Reyes A Pessina E Pujol P James G. Characteristics of patients with chronic kidney disease and Type 2 diabetes initiating finerenone in the USA: a multi-database, cross-sectional study. J Comp Eff Res (2023) 12(8):e230076. doi: 10.57264/cer-2023-0076 37387399 PMC10949885

[111] ElSayed NA Aleppo G Aroda VR Bannuru RR Brown FM Bruemmer D. 10. Cardiovascular disease and risk management: standards of care in diabetes-2023. Diabetes Care (2023) 46(Suppl 1):S158–90. doi: 10.2337/dc23-S010 PMC9810475 36507632

[112] de Boer IH Khunti K Sadusky T Tuttle KR Neumiller JJ Rhee CM. Diabetes management in chronic kidney disease: A consensus report by the American diabetes association (ADA) and kidney disease: improving global outcomes (KDIGO). Diabetes Care (2022) 45(12):3075–90. doi: 10.2337/dci22-0027 PMC9870667 36189689

[113] American Diabetes Association Professional Practice Committee. 11. Chronic kidney disease and risk management: standards of medical care in diabetes-2022. Diabetes Care (2022) 45(Suppl 1):S175–84. doi: 10.2337/dc22-S011 34964873

[114] Blonde L Umpierrez GE Reddy SS McGill JB Berga SL Bush M. American association of clinical endocrinology clinical practice guideline: developing a diabetes mellitus comprehensive care plan-2022 update. Endocr Pract (2022) 28(10):923–1049. doi: 10.1016/j.eprac.2022.08.002 35963508 PMC10200071

[115] Kidney Disease: Improving Global Outcomes (KDIGO) Diabetes Work Group. KDIGO 2022 clinical practice guideline for diabetes management in chronic kidney disease. Kidney Int (2022) 102(5S):S1–127. doi: 10.1016/j.kint.2022.06.008 36272764

[116] Goulooze SC Snelder N Seelmann A Horvat-Broecker A Brinker M Joseph A. Finerenone dose-exposure-serum potassium response analysis of FIDELIO-DKD phase III: the role of dosing, titration, and inclusion criteria. Clin Pharmacokinet (2022) 61(3):451–62. doi: 10.1007/s40262-021-01083-1 PMC8891103 34786651

[117] Agarwal R Joseph A Anker SD Filippatos G Rossing P Ruilope LM. Hyperkalemia risk with finerenone: results from the FIDELIO-DKD trial. J Am Soc Nephrol (2022) 33(1):225–37. doi: 10.1681/ASN.2021070942 PMC8763180 34732509

[118] Leon SJ Whitlock R Rigatto C Komenda P Bohm C Sucha E. Hyperkalemia-related discontinuation of renin-angiotensin-aldosterone system inhibitors and clinical outcomes in CKD: A population-based cohort study. Am J Kidney Dis (2022) 80(2):164–73.e1. doi: 10.1053/j.ajkd.2022.01.002 35085685

[119] Natale P Palmer SC Ruospo M Saglimbene VM Strippoli GF. Potassium binders for chronic hyperkalaemia in people with chronic kidney disease. Cochrane Database Syst Rev (2020) 6(6):CD013165. doi: 10.1002/14651858.CD013165.pub2 32588430 PMC7386867

[120] Zuo C Xu G. Efficacy and safety of mineralocorticoid receptor antagonists with ACEI/ARB treatment for diabetic nephropathy: A meta-analysis. Int J Clin Pract (2019) 29:e13413. doi: 10.1111/ijcp.13413 31464019

[121] Vaduganathan M Docherty KF Claggett BL Jhund PS de Boer RA Hernandez AF. SGLT-2 inhibitors in patients with heart failure: a comprehensive meta-analysis of five randomised controlled trials. Lancet (2022) 400(10354):757–67. doi: 10.1016/S0140-6736(22)01429-5 36041474

[122] Neuen BL Oshima M Perkovic V Agarwal R Arnott C Bakris G. Effects of canagliflozin on serum potassium in people with diabetes and chronic kidney disease: the CREDENCE trial. Eur Heart J (2021) 42(48):4891–901. doi: 10.1093/eurheartj/ehab497 34423370

[123] Neuen BL Oshima M Agarwal R Arnott C Cherney DZ Edwards R. Sodium-glucose cotransporter 2 inhibitors and risk of hyperkalemia in people with type 2 diabetes: A meta-analysis of individual participant data from randomized, controlled trials. Circulation (2022) 145(19):1460–70. doi: 10.1161/CIRCULATIONAHA.121.057736 35394821

[124] Provenzano M Jongs N Vart P Stefánsson BV Chertow GM Langkilde AM. The kidney protective effects of the sodium-glucose cotransporter-2 inhibitor, Dapagliflozin, are present in patients with CKD treated with mineralocorticoid receptor antagonists. Kidney Int Rep (2022) 7(3):436–43. doi: 10.1016/j.ekir.2021.12.013 PMC8897688 35257056

[125] Provenzano M PuChades MJ Garofalo C Jongs N D’Marco L Andreucci M. Albuminuria-lowering effect of dapagliflozin, eplerenone, and their combination in patients with chronic kidney disease: A randomized crossover clinical trial. J Am Soc Nephrol (2022) 33(8):1569–80. doi: 10.1681/ASN.2022020207 PMC9342643 35440501

[126] Rossing P Agarwal R Anker SD Filippatos G Pitt B Ruilope LM. Efficacy and safety of finerenone in patients with chronic kidney disease and type 2 diabetes by GLP-1RA treatment: A subgroup analysis from the FIDELIO-DKD trial. Diabetes Obes Metab (2022) 24(1):125–34. doi: 10.1111/dom.14558 PMC9293162 34580995

[127] Rossing P Agarwal R Anker SD Filippatos G Pitt B Ruilope LM. Finerenone in patients across the spectrum of chronic kidney disease and type 2 diabetes by glucagon-like peptide-1 receptor agonist use. Diabetes Obes Metab (2023) 25(2):407–16. doi: 10.1111/dom.14883 PMC10092103 36193847

[128] Green JB Mottl AK Bakris G Heerspink HJL Mann JFE McGill JB. Design of the COmbinatioN effect of FInerenone anD EmpaglifloziN in participants with chronic kidney disease and type 2 diabetes using a UACR Endpoint study (CONFIDENCE). Nephrol Dial Transpl (2023) 38(4):894–903. doi: 10.1093/ndt/gfac198 PMC10064838 35700142

[129] A Trial to Learn How Well Finerenone Works and How Safe it is in Adult Participants with Non-diabetic Chronic Kidney Disease. Available at: https://clinicaltrials.gov/ct2/show/NCT05047263 (Accessed 27 February 2023).

[130] Desai NR Navaneethan SD Nicholas SB Pantalone KM Wanner C Hamacher S. Design and rationale of FINE-REAL: A prospective study of finerenone in clinical practice. J Diabetes Complications (2023) 37(4):108411. doi: 10.1016/j.jdiacomp.2023.108411 36857997

